# Effects of Puerarin on the Prevention and Treatment of Cardiovascular Diseases

**DOI:** 10.3389/fphar.2021.771793

**Published:** 2021-12-07

**Authors:** Yan-Xi Zhou, Hong Zhang, Cheng Peng

**Affiliations:** ^1^ State Key Laboratory of Characteristic Chinese Medicine Resources in Southwest China, College of Pharmacy, Chengdu University of Traditional Chinese Medicine, Chengdu, China; ^2^ Library, Chengdu University of Traditional Chinese Medicine, Chengdu, China; ^3^ Institute of Interdisciplinary Medical Sciences, Shanghai University of Traditional Chinese Medicine, Shanghai, China

**Keywords:** puerarin, cardiovascular diseases, mechanisms, targets, clinical trials

## Abstract

Puerarin, an isoflavone glycoside derived from *Pueraria lobata* (Willd.) Ohwi, has been identified as a pharmacologically active component with diverse benefits. A large number of experimental and clinical studies have demonstrated that puerarin is widely used in the treatment of a variety of diseases. Among them, cardiovascular diseases (CVDs) are the leading cause of death in the world, and therefore remain one of the most prominent global public health concerns. In this review, we systematically analyze the preclinical investigations of puerarin in CVDs, such as atherosclerosis, cardiac hypertrophy, heart failure, diabetic cardiovascular complications, myocardial infarction, stroke and hypertension. In addition, the potential molecular targets of puerarin are also discussed. Furthermore, we summarize the clinical trails of puerarin in the treatment of CVDs. Finally, the therapeutic effects of puerarin derivatives and its drug delivery systems are overviewed.

## Introduction

Cardiovascular diseases (CVDs) remain a global health concern, as they are the number one cause of death in the world (Pardali et al., 2020). According to the World Heart Federation, 17.3 million people die from CVDs each year. By 2030, it is estimated that the number of deaths will reach 23.6 million (Smith et al., 2012). According to the American Heart Association’s methodology of projecting future care costs, by 2030, 40.5% of individuals in the United States will suffer from CVDs, with an estimated direct medical cost of $818 billion and indirect cost of $276 billion (Heidenreich et al., 2011). Therefore, effective treatment and the development of preventive intervention strategies to slow the progression of CVDs and reduce costs are momentous (Li et al., 2020). In terms of drug therapy, on the one hand, the demand for western medicine to control CVDs has not been met. On the other hand, natural product therapy has multiple targets and few adverse reactions. Thus, the latter has become an important research area for ameliorating CVDs (Hao et al., 2017; Varghese et al., 2018).

**FIGURE 1:**
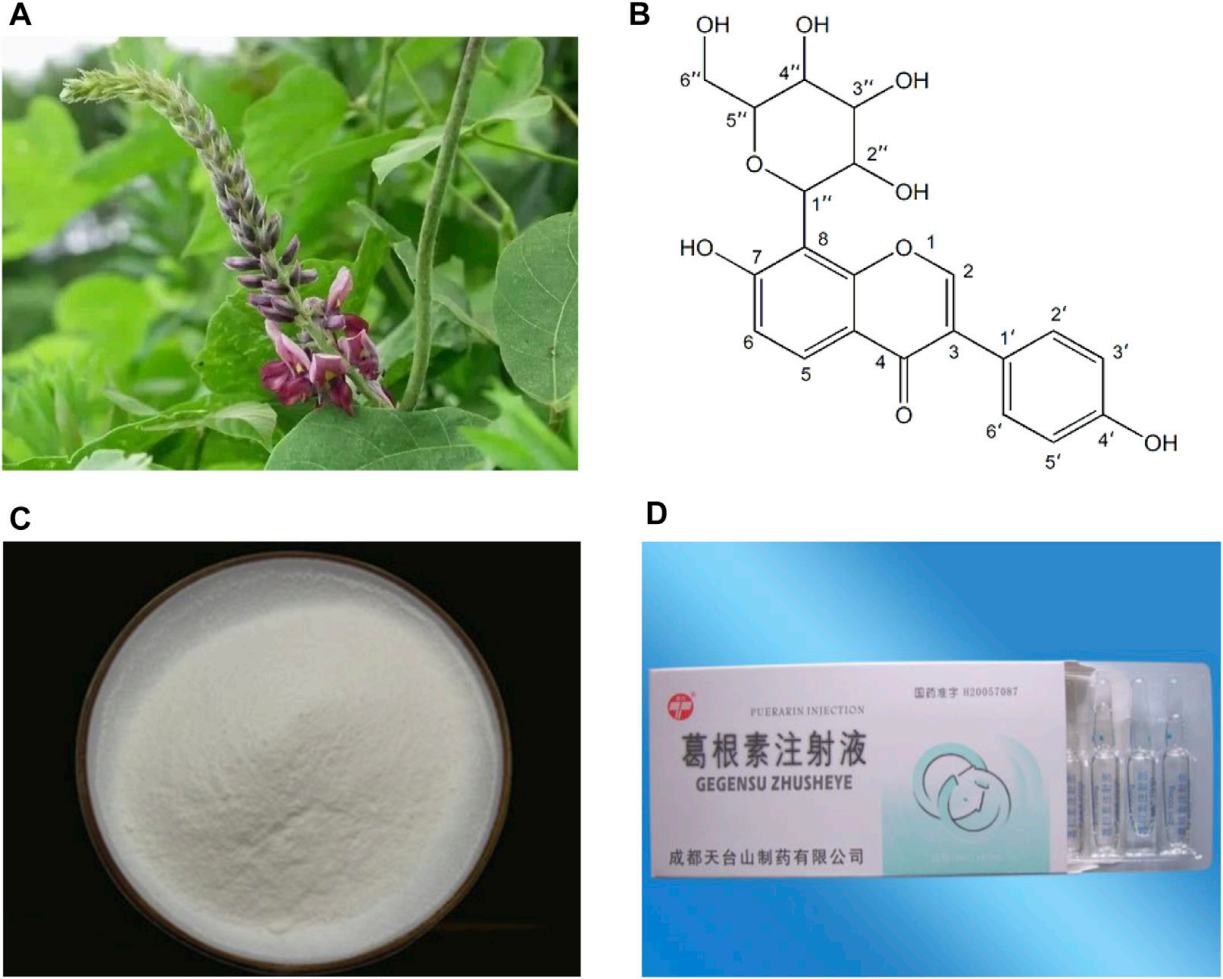
Pictures of *Pueraria lobata* (Willd.) Ohwi **(A)**, chemical structure of puerarin **(B)**, high purity puerarin crystalline powder **(C)** and Puerarin Injection **(D)**.

Kudzu root (Gegen in Chinese), the dried root of *Pueraria lobata* (Willd.) Ohwi (Figure 1A), is traditionally used as a traditional Chinese medicine (TCM) to promote blood circulation and increase blood flow (Zhang et al., 2013; Wong et al., 2011). Clinically, Gegen is mostly used in the prevention and treatment of CVDs and diabetes (Wong et al., 2011). Puerarin (C_21_H_20_O_9_; Figures 1B,C), an isoflavone glycoside, is the major bioactive ingredient of Gegen (Tian et al., 2016), which has been shown to be responsible for the pharmacological effects of Gegen in the cardiovascular system (Zhang et al., 2013). In addition, puerarin can also be isolated from several leguminous plants of the genus Pueraria, such as *Pueraria tuberosa* (Willd.) and *Pueraria thomsonii* Benth (Ahmad et al., 2020). Since the first isolation in the late 1950s, puerarin has been extensively studied for its effective and various pharmacological activities, including cardioprotection (Gao et al., 2006), vasodilation (Sun et al., 2007), anti-inflammation (Liu et al., 2013a), antioxidant (Jeon et al., 2020), attenuating insulin resistance (Zhang et al., 2010), neuroprotection (Zhao et al., 2020), alleviating pain (Wu et al., 2019), promoting bone formation (Yang et al., 2018a), inhibiting alcohol intake (Penetar et al., 2012) and anticancer (Hu et al., 2018). Experimental and clinical investigations have reported that puerarin is widely used in the treatment of CVDs, diabetes and its complications, Alzheimer’s disease, Parkinson’s disease, osteonecrosis, endometriosis and cancer (Zhou et al., 2014). In China, the injection form of puerarin, including Puerarin Injection (Figure 1D), Puerarin and Glucose Injection, as well as Puerarin and Sodium Chloride Injection, has been approved as a vasodilator for clinical treatment of coronary heart disease, angina, myocardial infarction (MI), retinal vein occlusion and sudden deafness (Zhang et al., 2013; Chen et al., 2018). In view of the pharmacological and therapeutic profile of puerarin in the cardiovascular system, this article reviews the cardiovascular actions of puerarin observed in experimental and clinical studies and the mechanisms underlying those effects. Moreover, the therapeutic utility of puerarin derivatives and its drug delivery systems are summarized.

## Cadiovascular Effects of Puerrarin

**FIGURE 2:**
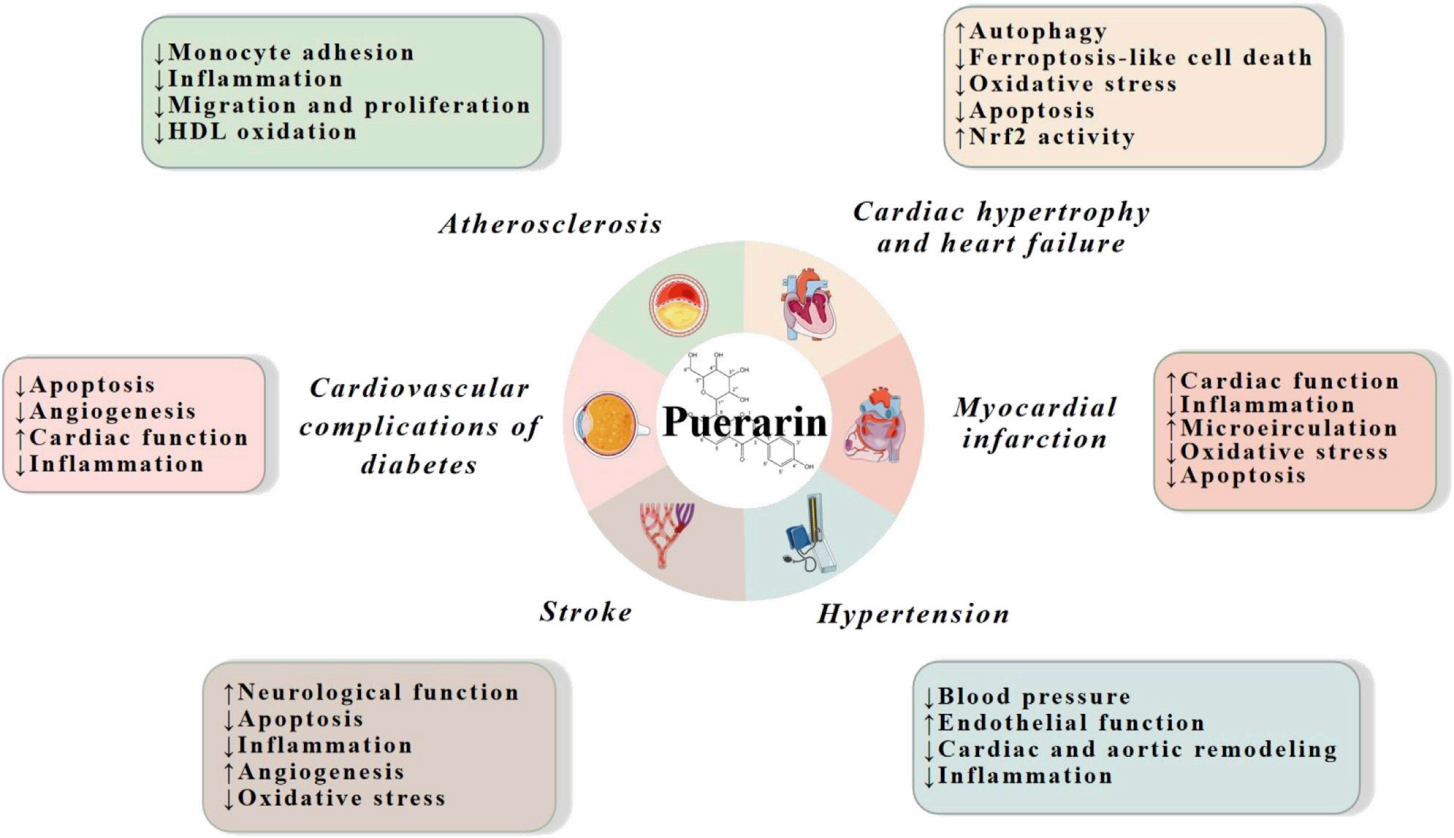
Cardiovascular actions of puerarin. HDL, high density lipoprotein; Nrf2, nuclear factor erythroid 2-related factor 2.

Multiple studies based on animal models and cell lines have demonstrated that puerarin has strong cardiovascular effects on a variety of CVDs, such as atherosclerosis, cardiac hypertrophy, heart failure, diabetic cardiovascular complications, myocardial infarction, stroke and hypertension. The mechanisms are mainly related to reduction of inflammation, oxidative stress and apoptosis, and promotion of cardiac, endothelial and neurological function (Figure 2).

### Atherosclerosis

Atherosclerosis, a chronic disease of the arterial wall, is the pathological basis of several diseases, such as cerebrovascular disease, coronary artery disease, acute coronary syndrome, MI and stroke (Weber and Noels, 2011). Recruitment of monocytes by activated endothelial cells to the vessel wall is an early event of atherogenesis (Mestas and Ley, 2008). The endothelium plays a vital role in controlling mass transportation, inhibiting thrombosis and modulating the growth and function of vascular smooth muscle cells (VSMCs) (Redmond et al., 2001; Kim et al., 2009). Numerous endothelial adhesion molecules including intercellular cell adhesion molecule-1 (ICAM-1), vascular cell adhesion molecule-1 (VCAM-1), selectin and integrin as well as extensive chemokines including interleukin-8 (IL-8) and monocyte chemoattractant protein-1 (MCP-1) mediate the adhesion of monocytes to vascular endothelium (Gerszten et al., 1999; Gerhardt and Ley, 2015; Wang et al., 2017). In this scenario, it has been shown that puerarin possesses the ability to modulate the expression of adhesion molecules and suppress monocyte endothelialial binding. *In vitro* puerarin (10 and 50 μM) decreased oxLDL-stimulated monocyte THP-1 adhesion to HUVECs and lowered the levels of adhesion-related proinflammatory chemokines (IL-8 and MCP-1) and endothelial adhesion molecules (ICAM-1 and VCAM-1). Furthermore, these protective effects were associated with activation of extracellular signal-regulated kinase 5/Kruppel-like factor 2 (ERK5/KLF2) pathway. Specifically, *in vitro* puerarin activated ERK5/KLF2 and then promoted the expression of endothelial nitric oxide synthase (eNOS) and thrombomodulin. *Ex vivo* the atherosclerotic lesions in both cross sections at aortic root and whole aorta of high fat-diet apolipoprotein E-deficient mice were markedly lessened by puerarin (100 mg/kg). Nevertheless, these alterations were reversed by XDM8-92 and BIX02189 (ERK5/KLF2 pathway inhibitors) (Deng et al., 2018).

Moreover, the migration and proliferation of VSMCs are important pathological components of atherosclerosis (Keramati et al., 2011; Yu et al., 2018). Intraperitoneal treatment of puerarin (50 and 100 mg/kg) facilitated re-endothelialization after balloon carotid denudation in rat and markedly prevented neointima formation. Besides, puerarin up-regulated the serum levels of nitric oxide (NO) and prostaglandin I_2_ (PGI_2_). *In vitro* puerarin strengthened late EPC and mature endothelial cell functions and inhibited the migration of VSMCs, suggesting that puerarin accelerated re-endothelialization by affecting vasodilator concentration and vascular cell functions (Cheng et al., 2013). In a rabbit model of fat diet-induced atherosclerosis, puerarin prevented the formation and development of atherosclerosis plaque and inhibited the reproduction and migration of VSMCs. Specifically, puerarin reduced the levels of total cholesterol, triglyceride and low density lipoprotein-cholesterol, while it increased high density lipoproteincholesterol production. Additionally, puerarin decreased whole blood viscosity and the expression of proliferation cell nuclear antigen and platelet-derived growth factor A protein levels of aorta (Bao et al., 2015). Besides, puerarin exerted less inhibitory effect on the high density lipoprotein oxidation induced by Cu^2+^ (Meng et al., 2004).

In addition to ameliorating vascular endothelial function and acting on smooth muscle cells, puerarin exerted anti-atherosclerotic activity through some other pathways. In a rat model of atherosclerosis, certain genes such as Pck1, Acaa2, DHCR7, Ascl1, Acad1, Acat1, and Nnat involved in lipid and energy metabolism were up-regulated, whereas they were down-regulated by puerarin. Besides, mitochondrial function in atherosclerosis rat aortas was meliorated by puerarin. Furthermore, peroxisome proliferator-activated receptor gamma (PPAR *γ*), retinoid X receptor and the corresponding downstream genes were up-regulated, whereas they were down-regulated by puerarin, suggesting that the therapeutic effect of puerarin on atherosclerosis is related to regulation of the PPAR pathway (Fu et al., 2014).

### Cardiac Hypertrophy and Heart Failure

The initial development of cardiac hypertrophy is an adaptive response of the heart to physiological and pathological stimuli (Haq et al., 2000). Physiological hypertrophy is characterized by normal or augmented contractile function, and normal structure and organization of cardiac tissue. Pathological hypertrophy is related to systolic dysfunction, interstitial fibrosis, cardiac structural remodeling and myocardial fibrosis (Oka et al., 2014; Kamo et al., 2015). Over time, pathological hypertrophy eventually results in heart failure, which is a leading cause of death in patients with CVDs in the world (Li et al., 2014). In cardiac tissues, the activation of the transforming growth factor-beta (TGF-β) signaling pathway is associated with the development of myocyte hypertrophy (Villar et al., 2009). It has been evidenced that the expression and biological activity of TGF-β have changed in hypertrophic cardiomyocytes (van Wamel et al., 2002). In a mouse model of angiotensin II (Ang II)-induced heart hypertrophy, puerarin (100 mg/kg) ameliorated heart hypertrophy through up-regulation of miR-15b/195 expression and inhibition of non-canonical TGF-β signal members (TGF-β-activated-kinase 1 and p38). Similar results were obtained in Ang II-treated primary ventricular cardiomyocytes of neonatal C57BL/6J mice (Zhang et al., 2016).

Moreover, a number of studies have demonstrated that the modulation of ROS production and the regulation of nuclear factor erythroid 2-related factor 2 (Nrf2) activation by puerarin are associated with oxidative stress in cardiomyocyte hypertrophy. *In vitro* puerarin at 50 and 100 μM prevented ERK1/2 and c-Jun N-terminal kinase 1/2 (JNK1/2) phosphorylation, activator protein-1 binding activity, ROS level and nicotinamide adenine dinucleotide phosphate (NADPH) oxidase activity by disrupting Rac1 activation and membrane translocation of oxidase subunits (Gang et al., 2015). Nrf2 is a key master switch controlling the expression of antioxidant and protective enzymes (Erkens et al., 2015). Puerarin at 50 mg/kg attenuated abdominal aortic constriction-induced cardiac hypertrophy in rats via regulation of Nrf2. It increased Nrf2 and reduced Kelch-like ECH-associated protein 1 in the myocardium. Moreover, it elevated the nuclear accumulation of Nrf2 in parallel and the expression of associated downstream proteins (heme oxygenase 1, glutathione S-transferase P1 and NAD(P)H:quinone oxidoreductase 1). *In vitro* puerarin at 100 μM exerted anti-hypertrophic effect and up-regulated the metabolic enzymes UDP-glucuronosyltransferase 1A1 and 1A9 levels through activation of Nrf2 in Ang II-treated cardiomyocytes (Zhao et al., 2018). Additionally, dysregulated autophagy is related to hypertrophic process (Nakai et al., 2007). In both aortic banding rats and isoprenaline-induced H9c2 cells, puerarin blocked iron overload and lipid peroxidation (Liu et al., 2018). In addition, it ameliorated autophagy and prevented cardiomyocyte hypertrophy and apoptosis. Moreover, it activated the phosphorylation of 5′-adenosine monophosphate kinase (AMPK), while it prevented the level of mammalian target of rapamycin (mTOR) target proteins S6 ribosomal protein and 4E-binding protein 1 *in vivo* (Liu et al., 2015a).

### Cardiovascular Complications of Diabetes

Diabetes Mellitus is an independent risk factor for CVDs (Yang et al., 2010a; Xie et al., 2020). The two predominant types of diabetes (type 1 and type 2) are both relevant to an increased risk of microvascular and macrovascular complications, the leading cause of morbidity and mortality in patients with diabetes (King et al., 2005; Forbes and Cooper, 2013). Major microvasculature defects evident in diabetes include diabetic retinopathy, diabetic kidney disease and diabetic neuropathy (Maric-Bilkan, 2017; Yang et al., 2017). Diabetic retinopathy is envisioned as a biomarker of generalized hyperglycemic damage in the microvasculature (Cheung and Wong, 2008). In addition to microvascular complications, macrovascular complications of diabetes include coronary heart disease, MI, stroke and peripheral vascular disease (Vinik and Flemmer, 2002; Chiha et al., 2012; Li et al., 2016; Ma, 2016). Additionally, both clinical and preclinical studies have demonstrated the existence of a specific diabetic cardiomyopathy independent of macrovascular complications (Davidoff et al., 2004; Huynh, Bernardo, McMullen and Ritchie, 2014). Clinically, diabetic cardiomyopathy is characterized by left ventricular hypertrophy and remodeling, and altered myocardial energy metabolism (Mizamtsidi et al., 2016). Increasing evidence suggests that puerarin is able to exert protective actions against these cardiovascular complications of diabetes.

Administration of puerarin at 140 mg/kg alleviated apoptosis of retinal pigment epithelial cells and lowered the mRNA expression of nitrotyrosine and inducible nitric oxide synthase (iNOS) and the protein expression of Fas/Fas ligand in Streptozotocin (STZ)-induced diabetic rats (Hao et al., 2012). Puerarin improved STZ-induced diabetic retinopathy by inhibiting the morphological changes of inner and outer nuclear layers via the down-regulation of vascular endothelial growth factor (VEGF) and hypoxia-inducible factor-1α (HIF-1α) expression (Teng et al., 2009). In retinal capillary endothelial cells (TR-iBRB2), puerarin (10, 25 and 50 μM) alleviated IL-1β-evoked leukocyte adhesion, the production of VCAM-1 and ICAM-1, mitochondrial dysfunction and cell apoptosis (Zhu et al., 2014). These results demonstrate that puerarin may be a potential therapeutic agent for the treatment of diabetic retinopathy.

In STZ-Nicotinamide-induced diabetic mice with permanent ligation of left anterior, puerarin promoted survival rate, restored cardiac function, augmented the expression and translocation of glucose transporter 4 and the phosphorylation of Akt, as well as decreased the expression of CD36 and PPAR α, suggesting that puerarin is able to improve cardiac dysfunction in diabetic patients after MI (Cheng et al., 2015). In diabetic rats treated with ischemia/reperfusion, puerarin (25, 50 and 100 mg/kg) decreased the myocardial infarct area and inflammatory response, while it elevated left ventricular developed pressure, NO level, caspase-3 activity, as well as the protein expression of phosphorylated-eNOS, VEGFA and Ang I, suggesting that puerarin may be an effective therapeutic candidate against cardiomyopathy in diabetic patients (Guo et al., 2018).

### MI

MI is a condition of acute myocardial necrosis caused by the imbalance of coronary blood supply and myocardial demand (Liu et al., 2016a). Previous studies have shown that MI is caused by a complex set of pathological processes including inflammatory cell infiltration, an increase in free radical formation, apoptosis, irreversible DNA damage, etc (Liu et al, 2017a; Raish, 2017). Several animal models have shown that puerarin is effective in suppressing MI through multiple mechanisms. In a mouse model of isoproterenol-induced MI, puerarin reversed the typical electrocardiogram abnormal changes (Li et al., 2018). Puerarin combined treatment with tanshinone IIA blocked the release of inflammatory cells, modulated the expression of inflammatory cytokines (IL-1β, IL-6, IL-10 and iNOS), prevented M1 macrophages expression in the early stage of inflammation and increased M2 macrophages expression in myocardial ischemia mice. In addition, the combination of tanshinone IIA and puerarin inhibited inflammation by reducing toll-like receptor 4 protein expression and promoting CCAAT/enhancer-binding protein-β protein expression. Moreover, the combined application ameliorated hemodynamics through promoting cardiac function, decreasing myocardial cell damage and collagen synthesis, as well as preventing myocardial fibrosis and heart remodeling (Gao et al., 2019). In a rat model of MI, puerarin lessened infarct area in the heart, induced angiogenesis and increased the production of VEGF, HIF-1α and eNOS (Zhang et al., 2006). In a dog model of acute MI, puerarin suppressed the platelet aggregation and the blood viscosity, promoted the opening and formation of coronary collateral circulation and attenuated myocardial infarct area (Liu et al., 2000).

Furthermore, timely reperfusion is critical for the salvage of ischemic myocardium. However, reperfusion can also induce injury (Heusch, 2013). Accumulating evidence indicates that puerarin plays a protective role in myocardial ischemia/reperfusion injury. In a rat model of MI reperfusion injury, puerarin inhibited MI and MI reperfusion, reduced creatine kinase and methylene dioxyamphetamine, while it elevated superoxide dismutase (SOD) (Wenjun et al., 2015). In H9c2 cells exposed to ischemia-reperfusion, puerarin hoisted cell viability, inhibited apoptosis, lowered the levels of lactate dehydrogenase, ROS, MDA and NADPH oxidase 2, as well as increased the expression of miR-21 and the contents of SOD, catalase and glutathione peroxidase. Nevertheless, these actions were reversed through transfection of an miR-21 inhibitor, suggesting that the protective effects of puerarin against myocardial ischemia/reperfusion injury through suppressing apoptosis and oxidative stress via miR-21expression (Xu et al., 2019).

### Stroke

Stroke is a leading cause of adult mortality and disability worldwide (Bonita et al., 2004; Sarikaya et al., 2015). It results from the loss of brain function due to an interruption of blood supply (Kalani et al., 2015), and can be compartmentalized into ischemic stroke and hemorrhagic stroke (Li et al., 2016). In addition, multiple mechanisms including inflammation, apoptosis, oxidative stress and other possible pathways are involved in the pathogenesis of stroke (Li et al., 2016). Intriguingly, it has been widely reported that puerarin is involved in neuroprotection, promotion of angiogenesis, anti-apoptosis, anti-oxidation and anti-inflammation, which is closely related to its prevention and treatment of stroke. In a rat model of middle cerebral artery occlusion, puerarin ameliorated neurological functions, attenuated infarct size, edema volume and autophagy in neurons, as well as augmented vascular remodeling after stroke (Xu et al., 2007; Wang et al., 2014; Wu et al., 2014; Hongyun et al., 2017). Moreover, it lessened the terminal deoxynucleotidyl transferase biotin-dUTP nick end labeling staining cells, enhanced the activation of erythropoietin activity, the expression of X-chromosome-linked inhibitor of apoptosis protein, the production of aspartate, glutamate, *γ*-aminobutyric acid (GABA) and taurine, the rate of glutamate/GABA, as well as lowered the levels of HIF-1α, iNOS, caspase-3 protein expression and tumor necrosis factor-α (TNF-α) mRNA expression (Xu et al., 2005; Xu et al., 2007; Chang et al., 2009; Gao et al., 2009). In another study, puerarin protected the brain neurocytes of rats in acute local ischemia brain injury by improving HSP70 expression. Meanwhile, it protected the brain neurocytes of rats in acute local cerebral ischemia/reperfusion injury by improving HSP70 expression and reducing the Fas expression (Pan and Li, 2008).

Combined treatment with catalpol and puerarin alleviated neurological deficiency induced by middle cerebral artery occlusion in rats. Moreover, catalpol and puerarin lessened infarct volume, water content in ischemic hemisphere, oxidative stress injury and apoptosis. Meanwhile, the combined treament promoted angiogenesis around the infarct of cortex and neurogenesis in the Hippocampal Dentate Gyrus, elevated regional cerebral blood flow and maintained vessel integrity (Liu et al., 2014a; Liu et al., 2017b). In another study, the combination of catalpol and puerarin restored neurological deficiency in motor, sense, balance and reflex, and protected neurovascular unit through suppression of oxidative stress and inflammatory responses and increase of certain protective factors in ischemic-reperfusion rats (Xue et al., 2016). *In vitro* in astrocytes after oxygen-glucose deprivation/reperfusion, puerarin reduced the level of pro-apoptosis factor and elevated the secretion of brain-derived neurotrophic factor (Wang et al., 2014). In cultured hippocampal neurons, it inhibited glutamate-stimulated apoptosis and necrosis (Xu et al., 2007). In human neutrophils *in vitro*, it suppressed respiratory bursts induced by formylMet-Leu-Phe (Chang et al., 2009). In oxygen-glucose deprivation-reoxygenation astrocytes, it inhibited apoptosis, modulated mRNA expression of abundant genes and impeded the olfactory transduction, janus kinase 2 and signal transducer and activator of transcription 3 pathways (Wei et al., 2017).

### Hypertension

Hypertension is one of the most common diseases afflicting humans throughout the world (Ettner et al., 2012). Long-term elevated or unstable blood pressure increases the risk of vascular complications, including stroke, ischemic heart disease, congestive heart failure, renal disease and ocular fundus abnormalities (Shi et al., 2019). Increasing evidence indicates that puerarin has anti-hypertensive effect in a variety of animal models of hypertension. In spontaneously hypertensive rats, puerarin (40 and 80 mg/kg, i. p.) reduced SBP, diastolic blood pressure and heart rate, enhanced the levels of NO and cGMP and the expression of phosphorylated eNOS protein, and lowered angiotensin II type 1-receptor (AT1) and caveolin-1 levels (Shi et al., 2019). Besides, puerarin (25, 50 and100 mg/kg, i. p.) lowered the mRNA expression of TGF-β1 and Smad3, while it raised the mRNA expression of Smad7 (Zhang et al, 2011a). Treatment with high dose puerarin (200 mg/kg) up-regulated AT1 and ACE2 mRNA expression in kidney, while administration of puerarin at 100 mg/kg reduced the expression of AT1 and ACE2 mRNA in heart. Thereby, AT1 and angiotensin-converting enzyme 2 (ACE2) might be a positive feedback regulation (Ye et al., 2008). In Ang II-infused hypertensive rats, puerarin decreased SBP, aortic and left ventricular weigh, lowered aortic medial thickness and myocardial cell surface area, restored endothelium-dependent relaxation, increased the protein expression of phosphor-eNOS (Ser1177), as well as reduced the expression of gp91phox, p22phox, TGF-β1 and VCAM-1 (Li et al., 2017a). In two-kidney, one-clip renal hypertensive rats, it reduced the mean of left ventricular end diastolic pressure and the weight ratio of left ventricular mass (left ventricle plus septum) to body weight, the values of the which were 65.24 and 13.12%, respectively. In addition, it decreased the contents of apelin-36 in the plasma and left ventricle, the expression of apelin and angiotensin receptor-like protein J receptor (APJ) mRNA in left ventricle, and the levels of apelin and APJ peptides in left ventricle, the values of the which were 24.21, 49.40, 27.40, 45.66, 65.36 and 62.87%, respectively (Jin, et al., 2009). Furthermore, it decreased apelin mRNA and protein expression in ischemic and non-ischemic kidneys, while it increased APJ mRNA and protein expression in non-ischemic kidneys, and these effects were augmented by the combination of puerarin and felodipine (Huang et al., 2013). In another study, puerarin decreased SBP, diastolic blood pressure and heart rate, attenuated renal interstitial fibrosis, lowered the level of Ang II and the mRNA expression of ACE and AT1, while it increased the level of Ang (1–7) and the mRNA expression of ACE2 and Mas in kidney of renovascular hypertensive rats (Bai et al., 2013).

### Others

In addition to the above-mentioned disorders, studies on experimental animals and cultured cells have shown that puerarin also plays a beneficial role in other CVDs. In chronic ischemia-induced vascular dementia rats, puerarin ameliorated hippocampal morphological changes, improved learning and memory function, promoted methyl-CpG binding protein 2 phosphorylation in the hippocampus, and lowered MDA, glutathione peroxidase and thiol production in the hippocampus and frontal cortex (Zhang et al., 2015; Wang et al., 2018). In hydrogen peroxide-treated SH-SY5Y cells, puerarin enhanced cell viability, scavenged ROS production and increased the production of Nrf2, FoxO1, FoxO3 and FoxO4 (Zhang et al., 2015). Additionally, it attenuated severe burn-stimulated acute myocardial injury *via* reduction of inflammation, neutrophil infiltration and oxidative stress in the heart of rats (Liu et al., 2015b). Puerarin at 2.5 mg/kg retarded CaPO_4_-induced aneurysm in mice. *In vitro* in TNF-α and CaPO_4_-stimulated macrophages, it reduced the Mmp9 mRNA expression, secreted protein production, as well as inhibited the production of ROS and the phosphorylation of inhibitor of I-κB, ERK and p38 (Tanaka et al., 2016). Besides, it lessened SBP and heart rate, eased pain, reduced mRNA and protein expression of P2X_3_ receptor (a member of P2X receptors family) in superior cervical ganglia, stellate ganglia and cervical dorsal root ganglia neurons, as well as down-regulated ATP-sensitive currents in superior cervical ganglia and cervical dorsal root ganglia neurons of myocardial ischemic rats (Liu et al., 2014b; Liu et al., 2014c). Combined administration of puerarin and Danshensu reduced ST elevation, ischemic size and serum contents of isoenzyme of creatine kinase, lactate dehydrogenase and malondialdehyde (MDA), while it up-regulated serum level of SOD in rats with acute ischemic myocardial injury (Wu et al., 2007).

## Signal Transduction Pathways of Puerarin in CVDS

**FIGURE 3:**
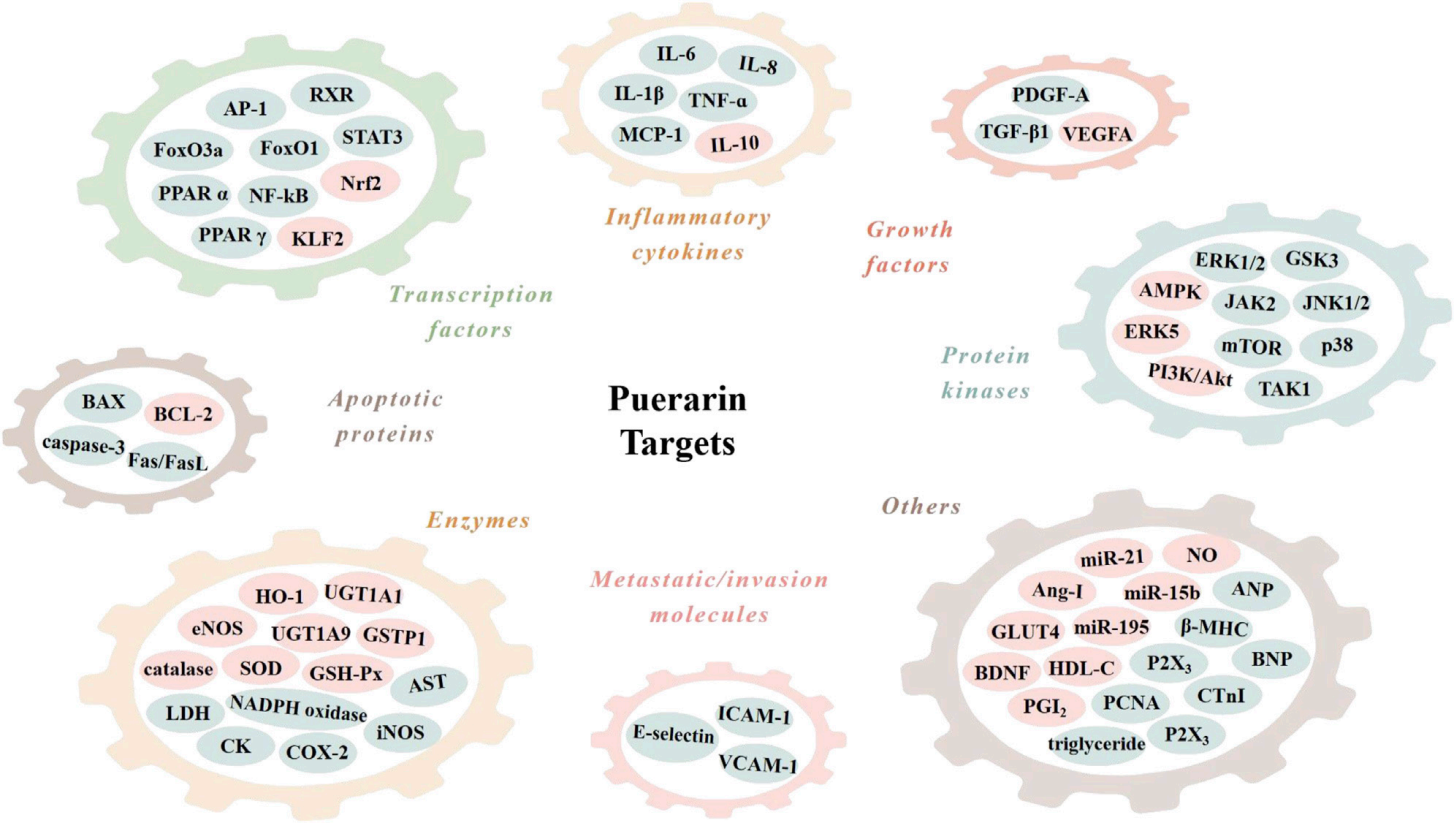
Molecular targets of puerarin. AMPK, adenosine monophosphate kinase; Ang-I, angiotensin I; ANP, atrial natriuretic peptide; AP-1, activator protein-1; AST, aspartate aminotransferase; BAX, BCL2-associated X protein; BCL-2, B cell lymphoma 2; BDNF; brain-derived neurotrophic factor; BNP, B-type natriuretic peptide; CK, creatine kinase; COX-2, cyclooxygenase-2; CTnI, cardiac troponin I; eNOS, endothelial nitric oxide synthase; ERK1/2, extracellular signal-regulated kinase 1/2; FasL, Fas ligand; FoxO1, forkhead box O1; GLUT4, glucose transporter 4; GSH-Px, glutathione peroxidase; GSK3, glycogen synthase kinase 3; GSTP1, glutathione S-transferase p 1; HDL-C, high density lipoproteincholesterol; HO-1, heme oxygenase 1; ICAM-1, intercellular cell adhesion molecule-1; IL-1β, interleukin-1β; iNOS, inducible nitric oxide synthase; JAK2, janus kinase 2; JNK1/2, c-Jun N-terminal kinase 1/2; KLF2, Kruppel-like factor 2; LDH, lactate dehydrogenase; MCP-1, monocyte chemoattractant protein-1; mTOR, mammalian target of rapamycin; NADPH oxidase, nicotinamide adenine dinucleotide phosphate oxidase; NF-κB, nuclear factor kappa B; Nrf2, nuclear factor erythroid 2-related factor 2; PCNA, proliferation cell nuclear antigen; PDGF-A, platelet-derived growth factor A; PGI_2_, prostaglandin I_2_; PPAR α, peroxisome proliferator-activated receptor α; RXR, retinoid X receptor; SOD, superoxide dismutase; STAT3, signal transducer and activator of transcription 3; TAK1, TGF-β-activated-kinase 1; TGF-β1, transforming growth factor-β1; TNF-α, tumor necrosis factor-α; UGT1A1, UDP-glucuronosyltransferase 1A1; VCAM-1, vascular cell adhesion molecule-1; VEGFA, vascular endothelial growth factor A; β-MHC, β-myosin heavy polypeptide.

The cardiovascular effects of puerarin are associated with its regulation of multiple targets in the CVD-related signal pathways (Table 1; Figure 3). Among them, reports on the effects on sodium (Na^+^), potassium (K^+^) and calcium (Ca^2+^) channels, nuclear factor kappa B (NF-κB), PI3K/Akt, BCL-2 and BAX are more concentrated.

**TABLE 1:** The involved molecular mechanims in the cardiovascular actions of puerarin.

Diseases	Cell lines and/or animal models	The specifific molecular mechanisms	Reference
Atherosclerosis	HUVECs and human THP-1 monocytes; high fat-diet apolipoprotein E-deficient mice	Activated the ERK5/KLF2 pathway	Deng et al. (2018)
	Rat EPCs, HUVECs and rat VSMCs; rat carotid balloon injury model	Enhanced the concentration of vasoactive substances; regulated vascular cell functions	Cheng et al. (2013)
	Fat diet-induced atherosclerosis in rabbits	Suppressed the expression of PCNA and PDGF-A	Bao et al. (2015)
	Vitamin D3 and cholesterol-induced atherosclerosis in rats	Regulated the PPAR pathway	Fu et al. (2014)
Cardiac hypertrophy and heart failure	HEK-293T cells and mouse primary cardiomyocytes; Ang II-induced heart hypertrophy in mice	Enhanced miR-15b/195 expression; inhibited non-canonical TGF-β	Zhang et al. (2016)
	Mouse primary cardiomyocytes; Ang II-induced cardiac hypertrophy in mice	Inhibited the AP-1 pathway	Gang et al. (2015)
	Abdominal aortic constriction-induced cardiac hypertrophy in rats	Promoted Nrf2 activity	Zhao et al. (2018)
	H9c2 cells; descending aortic banding-induced heart failure in rats	Inhibited ferroptosis	Liu et al. (2018)
	H9c2 cells; descending aortic banding-induced cardiac hypertrophy in rats	Regulated the AMPK/mTOR pathway	Liu et al. (2015a)
Cardiovascular complications of diabetes	STZ-induced diabetes in rats	Decreased the expression of peroxynitrite and iNOS	Hao, Wang, Ma and Yang, (2012)
	STZ-induced diabetic retinopathy in rats	Reduced the expression of VEGF and HIF-1α	Teng et al. (2009)
	TR-iBRB2 cells	Inhibited the expression of VCAM-1, ICAM-1, BAX and caspase-3; increased the expression of BCL-2	Zhu et al. (2014)
	STZ-Nicotinamide-induced diabetic mice after MI	Regulated the PI3K/Akt and PPARα pathway	Cheng et al. (2015)
	Ischemia/reperfusion-induced myocardial injury in diabetic rats	Promoted the VEGFA/Ang-I pathway; decreased caspase-3 activity	Guo et al. (2018)
MI	MI in rats	Induced the expression of VEGF and eNOS	Zhang et al. (2006)
	Acute MI in dogs	Improved microcirculation	Liu et al. (2000)
	H9c2 cells	Regulated the expression of miR-21	Xu et al. (2019)
Stroke	Rat cortical astrocytes; middle cerebral artery occlusion in rats	Reduced the expression of BAX and caspase-3; increased the expression of BCL-2 and the secretion of brain-derived neurotrophic factor	Wang et al. (2014)
	Rat primary hippocampal cells; middle cerebral artery occlusion and reperfusion in rats	Decreased the levels of aspartate, glutamate and γ-aminobutyric acid	Xu, Zheng, Zhou and Li, (2007)
	Middle cerebral artery occlusion in rats	Increased X-chromosome-linked inhibitor of apoptosis protein expression; reduced caspase-3 gene	Xu et al. (2005)
	Middle cerebral artery occlusion-induced brain infarction in rats	Inhibited the expression of HIF-1α, TNF-α, iNOS and active caspase-3	Chang et al. (2009)
	Acute cerebral ischemia and cerebral ischemia/reperfusion injury in rats	Increased HSP70 expression; decreased the Fas expression	Pan, & Li, (2008)
	Rat cortical astrocytes	Inhibited the olfactory transduction pathway and the JAK2/STAT3 pathway	Wei et al. (2017)
Hypertension	Spontaneous hypertension in rats	Enhanced the level of the phosphorylated eNOS protein	Shi et al. (2019)
	Spontaneous hypertension in rats	Decreased the expression of TGF-β1 and Smad3 mRNA; raised the expression of Smad7 mRNA	Zhang et al. (2011b)
	Spontaneous hypertension in rats	Regulated the expression of AT1 and ACE2 mRNA	Ye et al. (2008)
	Ang II-induced hypertension in rats	Increased the phosphorylation of eNOS at Ser 1177	Li et al. (2017a)
	Two-kidney, one-clip renal hypertension in rats	Modulated apelin/APJ system	Jin, et al. (2009)
Others	Vascular dementia in rats	Increased methyl-CpG binding protein 2 phosphorylation	Wang et al. (2018)
	SH-SY5Y cells; chronic ischemia-induced vascular dementia in rats	Enhanced the level of Nrf2, FoxO1, FoxO3 and FoxO4	Zhang et al. (2015)
	Severe burn-induced acute myocardial injury in rats	Reduced the TNF-α level and cardiac myeloperoxidase activity; abolished the activation of p38 MAPK	Liu et al. (2015b)
	CaPO4-Induced aneurysm in mice	Blocked the activity of I-κB, ERK and p38 and the level of ROS	Tanaka, Moriyama, Kawamura and Yamanouchi, (2016)
	Myocardial ischemic injury in rats	Regulated P2X3 receptor	Liu et al., 2014b; Liu et al., 2014c

ACE2, angiotensin-converting enzyme 2; AMPK, 5′-adenosine monophosphate kinase; Ang II, angiotensin II; AP-1, activator protein-1; APJ, angiotensin receptor-like protein J receptor; AT1, angiotensin II type 1-receptor; eNOS, endothelial nitric oxide synthase; EPCs, endothelial progenitor cells; ERK, extracellular signal-regulated kinase; FoxO1, forkhead box O1; HIF-1α, hypoxia-inducible factor-1α; HUVECs, human umbilical vein endothelial cells; ICAM-1, intercellular cell adhesion molecule-1; iNOS, inducible nitric oxide synthase; I-κB, inhibitor of NF-κB; KLF2, Kruppel-like factor 2; MAPK, mitogen-activated protein kinase; MI, myocardial infarction; mTOR, mammalian target of rapamycin; Nrf2, nuclear factor erythroid 2-related factor 2; PCNA, proliferation cell nuclear antigen; PDGF-A, platelet-derived growth factor A; PI3K, phosphoinositide 3-kinase; PPAR, peroxisome proliferator-activated receptor; ROS, reactive oxygen species; STAT3, signal transducer and activator of transcription 3; STZ, streptozotocin; TGF-β, transforming growth factor-beta; VCAM-1, vascular cell adhesion molecule-1; VEGF, vascular endothelial growth factor; VSMCs, vascular smooth muscle cells.

### Na^+^, K^+^ and Ca^2+^ channels

Na^+^ influx *via* slow Na^+^ channels may be associated with heart and brain hypoxic and ischemic damages (Ji and Wang, 1996). Inhibition of Ca^2+^-Na^+^ channels is considered to be an important indicator for the developments of potential anti-arrhythmia drugs (Zhang et al., 2003). Impaired K^+^ channel function in VSMCs is associated with a number of pathological conditions, including hypertension, ischemia/reperfusion, brain injury and diabetes. There are four major classes of K^+^ channels, including voltage-dependent K^+^, Ca^2+^-activated K^+^ (BK_Ca_), ATP-sensitive K^+^ (K_ATP_) and inward rectifier K^+^ (Kir) channels (Ko, 2008). Ca^2+^ influx causes an overall increase in cytoplasmic Ca^2+^ concentration resulting in vasoconstriction. Meanwhile, Ca^2+^ influx activates the local Ca^2+^ release event of ryanodine receptors, called Ca^2+^ sparks, which in turn activates nearby BK_Ca_ channels, causing hyperpolarized currents to counter vasoconstriction (Brenner et al., 2000). Accumulating evidence illustrates that puerarin can regulate the Na^+^ (Zhang et al., 2003), K^+^ (Miao et al., 2002; Findlay et al., 2008; Ravens and Cerbai, 2008) and Ca^2+^ (Guo, 2004) channels in cardiac myocytes, which may partly contribute to its anti-myocardial damage, anti-arrhythmic and anti-vasoconstrictive property.

In rat ventricular myocytes, puerarin exerted anti-myocardial ischemia and anti-arrhythmic action through preventing the cardiac Na^+^ channels (Zhang et al., 2003). In isolated rat heart, puerarin attenuated myocardial ischemia and reperfusion injury by suppressing mitochondrial permeability transition pore opening, activating the mitochondrial K_ATP_ channel (Gao et al., 2005; Gao et al., 2006) and BK_Ca_ channel opening, and elevating protein kinase C (Gao et al., 2007). In another study, puerarin at 2.4 mM time-dependently lowered L-type Ca^2+^ current of rat ventricular myocytes. Moreover, it increased the current-voltage curve of Ca^2+^ current, suggesting that puerarin provided protective effects on rat ventricular myocytes against cardiac damage and arrhythmia by suppression of L-type Ca^2+^ channel (Guo, 2004). Both in rat ventricular cells and *Xenopus* oocytes, puerarin restrained the Kir channel current (Zhang et al., 2011a). Additionally, the protective effect of puerarin on rat isolated cardiomyocytes against H_2_O_2_-stress was related to prevention of mitochondrial permeability transition pore opening and activation of mitochondrial BK_Ca_ channels (Yang et al., 2008). Puerarin exerted a vasorelaxant action on rat basilar artery rings pre-contracted by U46619. The mechanism was related to two pathways, one was an endothelium-dependent pathway associated with the production of NO, and the other was an endothelium-independent pathway involving the opening of K^+^ channels (Deng et al., 2012). Furthermore, puerarin at 0.1–1000 μM concentration-dependently exerted the vasodilatory action on noradrenaline bitartrate-induced rat thoracic aortac rings by activating large-conductance BK_Ca_ channels, especially BK-*α*+*β*1 channels (Sun et al., 2007). Puerarin at 50, 150 and 450 μM restrained the contraction of rat aortic rings stimulated by phenylephrine or KCl, and its action was in an endothelium-dependent manner. Meanwhile, the anti-vasoconstrictive effect of puerarin was exerted by triggering extracellular Ca^2+^ influx (Yan et al., 2009).

In the above studies, the specific ion channels that puerarin acts on were investigated by treatment with various channel inhibitors, such as 5-hydroxydecanoate (the mitochondrial specific K_ATP_ channel blocker) (Gao et al., 2005; Gao et al., 2006), paxilline and iberiotoxin (blockers of the BK_Ca_ channel) (Gao et al., 2007; Sun et al., 2007; Yang et al., 2008), barium (an open-channel inhibitor of Kir) (Zhang et al., 2011b) and three other K^+^ channel inhibitors, glibenclamide, tetraethylammonium and 4-aminopyridine (Yan et al., 2009; Deng et al., 2012).

### NF-κB

The transcription factor NF-κB, a nuclear protein, was initial discovered by David Baltimore’s group in 1986 (Sen and Baltimore, 1986a; Sen and Baltimore, 1986b). NF-κB is a family of structurally related and functionally conserved dimeric proteins that consist of five members trapped in the cytoplasm under non-activated conditions: c-Rel, RelA (p65), RelB, p50/p105 and p52/p100 (Liu et al., 2013b; Ma and Hottiger, 2016). NF-κB binds to target DNA elements in the nucleus and positively regulates gene transcription involved in inflammatory and immune responses, cell growth control and apoptosis (Baldwin, 2001). Increasing evidences suggest that the cardiovascular regulatory activity of puerarin may involve the regulation of the NF-κB pathway.

In Dahl salt-sensitive hypertensive rats, puerarin ameliorated vascular insulin resistance and attenuated cardiovascular remodeling involving the suppression of NF-κB/JNK and ERK1/2 pathway (Tan et al., 2017). In Sprague-Dawley (SD) rats, administration of puerarin alleviated ischemia/reperfusion injury stimulated by 90 min of middle cerebral artery occlusion and followed by 2, 6, 12, 24 and 72 h reperfusion *via* suppressing the activation of NF-κB (Ding et al., 2007). In STZ-induced diabetic rats and in rat embryonic cardiomyoblast-derived H9c2 cells injured by high glucose *in vitro*, puerarin prevented diabetic cardiomyopathy through inhibiting inflammation via suppression of NF-kB activation (Yin et al., 2019). Moreover, it prevented Ang II-stimulated cardiac hypertrophy in C57BL/6J mice and in cultured cardiomyocytes via the redox-sensitive ERK1/2, p38 and NF-κB pathways (Chen et al., 2014). In Kunming mice, it inhibited isoprenaline-induced myocardial fibrosis through inhibiting TGF-β1 expression via activation of PPAR α/γ and subsequent blockade of NF-κB in myocardial tissue (Chen et al, 2012a). In addition, by reducing NF-κB activation, puerarin inhibted the inflammatory response in a rabbit model of atherosclerosis *in vivo* (Ji et al., 2016a) and in peripheral blood mononuclear cells of patients with stable angina pectoris *in vitro* (Yang et al., 2010b).

### PI3K/Akt

PI3K play a vital role in the pathogenesis of CVDs *via* modulating several basic cellular functions, such as cell migration, translational responses and cell survival, and subsequently regulating certain essential biologic processes, such as vascular homeostasis, metabolism and thrombogenicity (Eisenreich and Rauch, 2011). PI3K mediates key signaling pathways through several downstream molecules, such as Akt, mTOR, 3-phosphoinositide-dependent kinase 1 and glycogen synthase kinase 3 (Cantley, 2002; Wymann et al., 2003). A large body of studies indicate that the PI3K/Akt signaling pathway may be involved in the cardiovascular protective activity of puerarin.

In a rat model of MI, puerarin enhanced serum NO content by increase of eNOS expression and Akt pathway (Zhang et al., 2008). In aortic banding mice, oral treatment with puerarin eased the progression of cardiac hypertrophy and ameliorated cardiac function by reduction of PI3K/Akt and JNK signaling (Yuan et al., 2014). Both in transgenic TG (fli1:EGFP) zebrafish and in cultured human endothelial cells, puerarin attenuated Aß40-induced vascular dysfunction *via* inhibition of NADPH oxidase-derived ROS overproduction and activation of the PI3K/Akt/eNOS pathway (Lu et al., 2014a). In neonatal rat cardiomyocytes isolated from the ventricular heart of 1–2 days old SD rats, puerarin retarded myocardial ischemia/reperfusion injury through reducing autophagy *via* the Akt signaling pathway (Tang et al., 2017). In daunorubicin-treated H9c2 cells, puerarin inhibited cell apoptosis by activating the PI3K/Akt pathway *via* the suppression of intracellular Ca^2+^ influx (Li et al., 2017b). In EA. hy926 endothelial cells, puerarin stimulated the phosphorylation of eNOS and the production of NO by provoking estrogen receptor-mediated PI3K/Akt- and Ca^2+^/calmodulin-dependent kinase II/AMPK-dependent signaling pathway (Hwang et al., 2011). Furthermore, it inhibited the onset of endothelial progenitor cell (EPC) senescence and promoted EPC proliferation *in vitro*, which might be mediated by the activation of telomerase *via* the PI3K/Akt pathway (Zhu et al., 2008). It was effective in abating osteoblastic differentiation of calcifying VSMCs as indicated by reduced alkaline phosphatase activity, osteocalcin secretion and Runx2 expression, which was related to targeting the estrogen receptor-mediated PI3K/Akt signaling pathway (Lu et al., 2014b). Additionally, it attenuated the neurocyte apoptosis and protected against cerebral ischemia/reperfusion injury in rats induced by Longa’s suture method possibly through the activation of the PI3K/Akt pathway (Han et al, 2012a). Combined treatment with catalpol and puerarin protected primary brain vascular endothelial cells from ischemic injury by promoting HIF-1α *via* activation of ERK and PI3K/Akt (Liu et al., 2017a). In the above studies, the PI3K inhibitor(s), such as LY294002 (Han et al, 2012b; Lu et al., 2014a) and wortmannin (Zhu et al., 2008), or the Akt inhibitor, such as API-2 (Tang et al., 2017) and 1L-6-hydroxymethyl-chiro-inositol 2-(R)-2-O-methyl-3-O-octadecylcarbonate (Lu et al., 2014b), was/were used to confirm that PI3K/Akt pathway does play a role in the cardiovascular effect of puerarin.

### BCL-2 and BAX

BCL-2 family proteins, including both pro-life (e.g., BCL-2, BCL-X_L_) and pro-death (e.g., BAX, BAK) members, are the key regulators and executors of the intrinsic apoptosis pathway (van Delft and Huang, 2006; Singh et al., 2019). Apoptosis is a mechanism of programmed cell death and plays an important role in human health and disease (Volkmann et al., 2014). BAX and BAK regulate a cell to its programmed death by permeabilizing the mitochondrial outer membrane and then initiating the caspase cascade (Edlich, 2018). *In vivo* and *in vitro* studies suggest that the effect of puerarin on cardiovascular cell apoptosis may be related to its regulation of the expression of BCL-2 and BAX.

Puerarin attenuated apoptosis in rat middle cerebral artery occlusion brain by elevating the levels of cleaved caspase-3 and BAX and the ratio of BAX/BCL-2. Furthermore, puerarin inhibited apoptosis in primary cultured cortical rat astrocytes induced by oxygen-glucose deprivation/reperfusion injury *via* down-regulation of BAX level and BAX/BCL-2 rate and up-regulation of BCL-2 level and brain-derived neurotrophic factor expression (Wang et al., 2014). In CoCl_2_-damaged PC12 cells, combined administration of catalpol and puerarin inhibited apoptosis involving increase of BCL-2 level and decrease of BAX/BCL-2 ratio and caspase-3 level (Liu et al., 2014a). Puerarin treatment caused the same change trend on BCL-2 level and caspase-3 activity in rat H9c2 cardiomyocytes under H/R conditions (Xu et al., 2019). In rat pulmonary artery smooth muscle cells, puerarin induced apoptosis as indicated by down-regulated mitochondrial membrane potential and BCL-2 protein expression, cytochrome c release from the mitochondria, increased BAX protein expression and caspase-9 activation (Zhang et al, 2011c; Chen et al., 2012b). In subarachnoid hemorrhage mice, puerarin abated the neurological deficits, cerebral edema and blood-brain barrier disruption. In addition, puerarin elevated the ratio of BCL-2/BAX and the production of SOD and Sirt3, while it repressed the levels of cleaved caspase-3 and ROS (Zhang et al, 2019a). In SD rats, puerarin enhanced the learning-memory ability after global cerebral ischemia/reperfusion by attenuating apoptosis via elevation of BCL-2 expression (Wu et al., 2009).

## Clinical Trials of Puerarin in CVDS

In addition to the cardiovascular benefits observed in cell-based and animal models, puerarin also exerted protective effects on patients with CVDs (Table 2). An open, controlled, randomized and parallel-group comparison trail (http://clinicaltrials.gov/, NCT02254655) on 119 subjects with a definite diagnose of active rheumatoid arthritis demonstrated that 24 weeks of intravenous treatment with puerarin reduced carotid intima-media thickness and its action might be related to the amelioration of insulin resistance, indicating that puerarin might be a potential therapeutic candidate against cardio-metabolic comorbidities in patients with active rheumatoid arthritis (Yang et al., 2018b). A clinical trail was carried out among 120 coronary artery disease patients with stable angina pectoris. Both treatment and control groups were treated with nitrate, Ca^2+^ antagonists, β-receptor blockers, statins and aspirin. Patients in the treatment group were given 28 consecutive days of combinative injection with puerarin. At the end of the study, the treatment group was better than the control group in terms of the total effective rate, the duration of angina pectoris, the improvement of the ST segment depression of electrocardiogram, the number of abnormal leads and the scores of Seattle angina questionnaire. Furthermore, there was a elevation of EPCs and NO levels and reduction of endothelin 1 (ET-1) expression and the levels of serum hypersensitive C-reactive protein, TNF-α and IL-6, and these alterations were more obvious in the treatment group than those in the control group (Zhang et al., 2019b). A study involving 388 patients with angina pectoris was conducted and it was found that the total efficacy of puerarin injection group and danshen injection group was 88.14% (171/194) and 61.86% (120/194), respectively. In addition, no obvious adverse reactions were noticed in both groups (Luo et al., 2012). A study carried out on 79 patients with anterior acute MI showed that the infarct size on the 28th day and the contents of granulocyte colony-stimulating factor, matrix metalloproteinase-9, IL-6 and TNF-α on the 7th day were reduced in the puerarin group (Xiao et al., 2005). A 2-weeks randomized controlled trial was conducted on 61 patients with acute MI. The results indicated that puerarin reduced plasma concentration of free fatty acids, suppressed inflammation and stabilized atherosclerotic plaques, and it was involved in the reduction of myocardial infarct size (Xiao et al., 2004). In another clinical trail, puerarin was demonstrated to be an effective therapeutic agent for cerebral vasospasm in patients after aneurysm subarachnoid hemorrhage. Puerarin treatment elevated the plasma concentration of NO, ET-1 and 6-keto-prostaglandin F1α, the mean velocity of middle cerebral artery, and the Glasgow outcome scale at discharge, while it reduced the plasma level of thromboxane B_2_ and the incidence of cerebral vasospasm (Wang et al., 2012). Recently, a randomized, double-blind, placebo-controlled, 2-way crossover trial (http://clinicaltrials.gov/, NCT03676296) on the effect of puerarin supplementation on CVD risk factors in men has been completed. The expected results are to obtain the short-term effect of puerarin on CVD risk factors in men, and provide evidence for the effect of puerarin on other potentially relevant risk factors, such as blood pressure, fasting blood glucose and testosterone, as well as some related biomarkers.

**TABLE 2:** Clinical trails of puerarin in treating CVDs.

Patients	Dose	Duration	Route of administration	Outcome measures	Reference
119 patients with a definite diagnose of active rheumatoid arthritis	400 mg/d	24 weeks	i.v.	CIMT and HOMA-IR	Yang et al. (2018b)
120 coronary artery disease patients with stable angina pectoris	400 mg/d	4 weeks	i.v.	SAQ, EPCs, NO, ET-1, TNF-α, hs-CRP, IL-6	Zhang et al. (2019b)
388 patients with angina pectoris	400 mg/d	4 weeks	i.v.	Total efficacy	Luo et al. (2012)
79 patients with anterior acute MI	500 mg/d	2 weeks	i.v.	Ideker QRS score, plasma G-CSF, MMP-9, IL-6 and TNF-α	Xiao et al. (2005)
61 patients with acute MI	500 mg/d	2 weeks	i.v.	Ideker QRS score, plasma FFA, MMP-9 and CRP	Xiao et al. (2004)
54 patients with aneurysm subarachnoid hemorrhage	500 mg/d	2 weeks	i.v.	Plasma NO, ET-1, 6-K-PGF1α and TXB2	Wang et al. (2012)

6-K-PGF1α, 6-keto-prostaglandin F1α; CIMT, carotid intima-media thickness; CVDs, cardiovascular diseases; EPCs, endothelial progenitor cells; ET-1, endothelin 1; FFA, free fatty acids; G-CSF, granulocyte colony-stimulating factor; HOMA-IR, homeostasis model assessment of insulin resistance; hs-CRP, hypersensitive C-reactive protein; IL-6, interleukin-6; MI, myocardial infarction; MMP-9, matrix metalloproteinase-9; NO, nitric oxide; SAQ, Seattle angina questionnaire; TNF-α, tumor necrosis factor a; TXB_2_, thromboxane B_2_.

A Cochrane review revealed that there was no notable difference between the puerarin group and the control group in death or dependence of patients with acute ischemic stroke (OR, 0.81; 95% CI, 0.35–1.87) (Tan et al., 2008). Similarly, another Cochrane review concluded that there was insufficient evidence to assess the clinical efficacy of puerarin on the survival or dependence of patients with ischemic stroke (Liu et al., 2016b). However, a recent meta-analysis showed that the clinical effective rate of puerarin injection in the treatment of acute ischemic stroke was higher than the control drugs (RR 1.22, 95% CI 1.17 to 1.28, *p* < 0.001). Moreover, puerarin injection evidently ameliorated the neurological deficit (MD -3.69, 95% CI -4.67 to -2.71, *p* < 0.001). The hemorheology index and fibrinogen of puerarin injection were significantly lower than those of the control group. Collectively, it was suggested that puerarin injection might be more effective in the clinical treatment of acute ischemic stroke with relative safety. Nevertheless, the current evidence was not adequate in virtue of the poor methodological quality and short of sufficient safety data. The adverse reactions of puerarin injection in curing acute ischemic stroke included nausea, vomiting, and other mild gastrointestinal discomfort, facial flushing, dizziness and allergic reaction (Zheng et al., 2017).

## The Derivatives and Drug Deliery Systems of Puerarin

**FIGURE 4:**
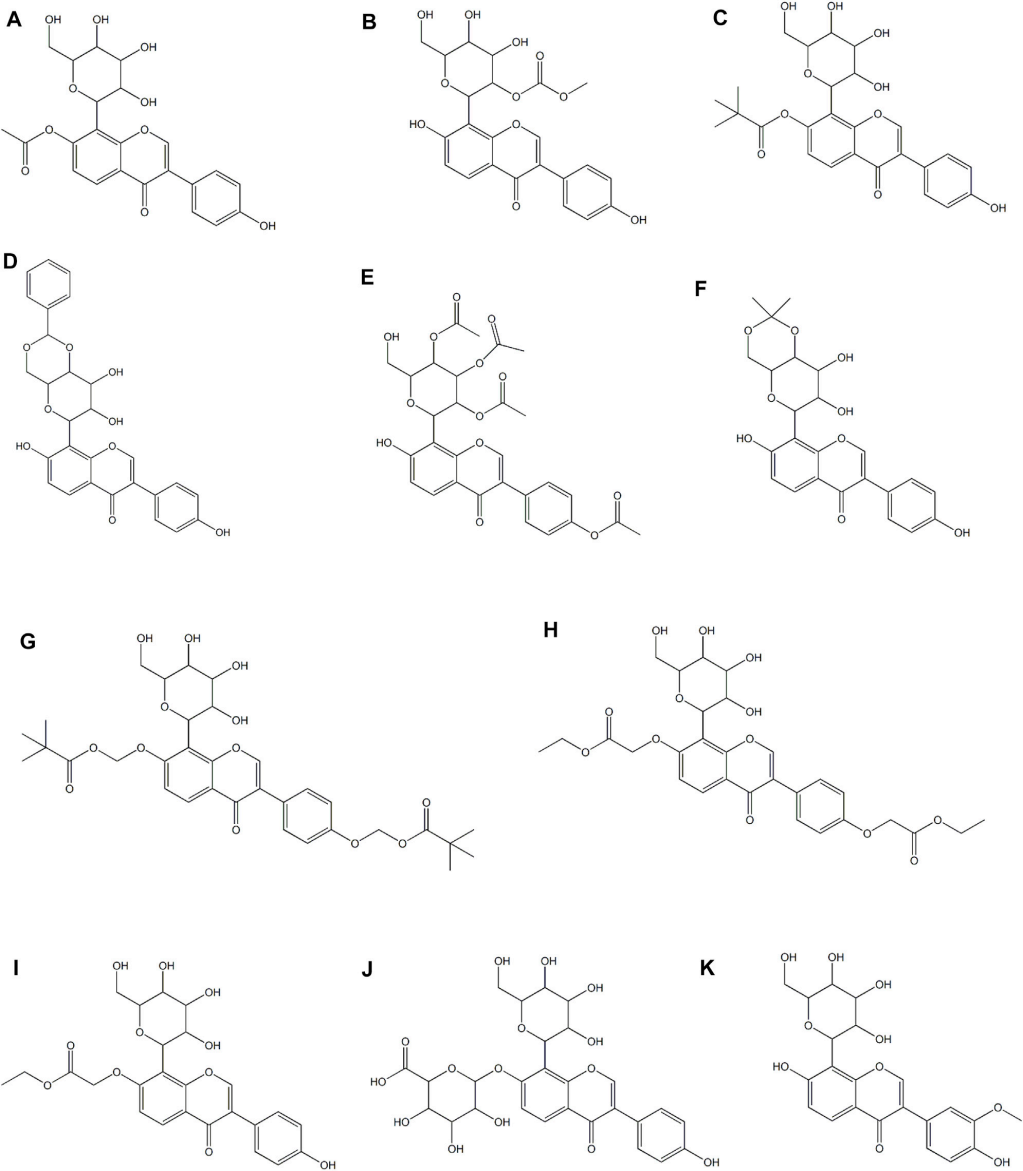
Derivatives of puerarin in treating CVDs.

The chemical structure of puerarin leads to low solubility and permeability, which results in poor oral absorption and low bioavailability. Simply increasing the dose will not effectively improve the bioavailability, and may lead to toxicity and side effects (Liu et al, 2016c). In view of this situation, a large number of studies are focused on developing derivatives or drug delivery systems to improve bioavailability and expand clinical applications. Among them, strategies include nanotechnologies and various preparation technologies (Zhang, 2019). In this section, only the derivatives and drug delivery systems of puerarin designed for cardiovascular applications are included. In a study, 7 puerarin derivatives (Figures 4A–G) were evaluated for their cardioprotective effect in the model of myocardial I/R injury of Langendorff isolated rat heart and the model of pituitrin-stimulatd SD rat myocardial ischemia, suggesting that the acylated modification of phenolic hydroxyl at C-7 in the puerarin molecule promoted the cardioprotective effect against myocardial ischemia and reperfusion injury, and the effect on the activity was related to the level of the bulk of the acylating agent (Feng et al., 2010). Puerarin derivative P1-EA (Figure 4H) and P2-EA (Figure 4I) were formed by substitution of the original phenolic hydroxyl in the puerarin molecules, which had higher lipophilicity and thus better permeabilized through the blood-brain barrier as compared to puerarin. It was resulted in an enhancement of protective activity against cerebral ischemic damage in a mouse model of ischemia/reperfusion-evoked dementia. Specifically, these two derivatives showed stronger suppression of inflammation (iNOS activity) and elevation of brain cortex Ca^2+^-Mg^2+^-ATPase activity (Ji et al., 2016b). Puerarin-7-*O*-glucuronide (Figure 4J), a water-soluble metabolite of puerarin, lessened intracellular superoxide level and improved total SOD activity, glutathione/glutathione disulfide ratio and total anti-oxidant capacity in xanthine oxidase/xanthine-induced neonatal rat cardiomyocytes. Similarly, it lowered superoxide production, NADPH oxidase activity and the expression of its subunits (p22^phox^ and p47^phox^) in Ang II-stimulated neonatal rat cardiomyocytes. In addition, it lowered hypertrophic changes in Ang II-induced neonatal rat cardiomyocytes. Collectively, puerarin-7-*O*-glucuronide inhibited Ang II-induced cardiomyocyte hypertrophy *in vitro via* reduction of oxidative stress (Hou et al., 2017). In a rat model of cerebral ischemia/reperfusion injury, 3′-methoxy puerarin (Figure 4K) improved the cerebral tissue pathologic changes, enhanced the level of PGI_2_ in cerebral tissue and the activity of plasma tissue-type plasminogen activator, and lowered the activity of plasma plasminogen activator inhibitor and the mRNA expression of ET-1 in cerebral tissue (Zhao et al., 2007). Moreover, it ameliorated the symptoms of neurological deficit, lowered the infarct volume and the level of MDA in cortex, and up-regulated SOD activity (Han et al., 2009). Another study reported that 3′-methoxy puerarin protected acute cerebral infarction by inhibiting the levels of both excitatory amino acid (aspartate and glutamate) and inhibitory amino acid (taurine and GABA), and thereby regulated the dynamic changes of amino acids (Han et al, 2012b).

Nanoparticles containing puerarin and hydroxypropyl-β-cyclodextrin inclusion complex lowered infarction volume, reduced the brain infiltration of inflammatory cells and the neuronal pyknosis and karyolysis, alleviated cell apoptosis, and improved electroencephalogram in middle cerebral artery occlusion/reperfusion model rats (Tao et al., 2013). The drug concentrations in blood and brain for puerarin loaded poly (butylcyanoacrylate) nanoparticles (PBCN) were both higher than those for the free drug, suggesting the permeability of puerarin loaded PBCN coated with polysorbate 80 on the blood-brain barrier was higher than that of puerarin. Additionally, compared to free drug, intravenous administration of puerarin loaded PBCN showed greater protective activity on focal cerebral ischemic injury in rats, and its action was related to raising body weight and reducing neurological deficit scores, brain water content and the infarct volume (Zhao et al., 2013). In another study, a puerarin nanoparticle based on glycyrrhetinic acid-PEG-PBLA was synthesized and its protective effect against liver ischemia/reperfusion injury was evaluated *in vivo*. Synthesized glycyrrhetinic acid-PEG-PBLA improved the water solubility, bioactivity and litter half-life of puerarin and augmented the liver-targeted drug delivery. Moreover, pretreatment with puerarin/glycyrrhetinic acid-PEG-PBLA complex ameliorated liver dysfunction and oxidative stress via the toll-like receptor 4/NF-κB pathway, thus alleviating liver ischemia/reperfusion injury (Xiao et al., 2018). In a rat model of acute myocardial ischemia stimulated by coronary artery ligation, puerarin PEG-PLGA micelles obviously promoted the uptake of puerarin by cardiomyocytes, augmented the anti-acute myocardial ischemia activity of puerarin and lowered its dosage (Liu et al., 2019). RGD modified and PEGylated solid lipid nanoparticles loaded with puerarin (RGD/PEG-puerarin-SLN) elevated the bioavailability of puerarin, prolonged the retention time *in vivo* and increased the protective activity against acute myocardial ischemia injury. The areas under the concentration-time curve augmented from 52.93 (mg/mL•h) for free puerarin to 176.5 (mg/mL•h) for RGD/PEG-puerarin-SLN. T_1/2_ elevated from 0.73 h for free puerarin to 2.62 h for RGD/PEG-puerarin-SLN. Compared to other puerarin formulations, RGD/PEG-puerarin-SLN showed higher drug concentration in heart and plasma. Furthermore, the myocardial infarct size of RGD/PEG-puerarin-SLN was the lowest among all the formulations in acute MI model rats. The infarct size of RGD/PEG-puerarin-SLN, PEG-puerarin-SLN, puerarin-SLN and free puerarin was 6.2, 18.1, 28.3 and 36.0%, respectively (Dong et al., 2017). Similarly, the *in vivo* myocardial infarct therapy efficiency of puerarin-prodrug and tanshinone co-loaded SLN, puerarin-prodrug-SLN and tanshinone-SLN were evaluated in MI rats, which was 17 ± 1.9%, 31 ± 1.6% and 40 ± 2.2%, respectively. Additionally, SLN encapsulation attenuated the cytotoxicity of the drug and was a safer system. These results suggested that puerarin-prodrug contained, double drugs co-loaded SLN might be a candidate drug delivery system for cardioprotective drugs for the treatment of MI (Guo et al., 2019).

In addition to puerarin derivatives and its drug delivery systems, the modified crystal forms of puerarin also have better absorption and higher plasma drug concentration than puerarin. Puerarin-V, a new advantageous crystal form of puerarin, suppressed the typical ST segment depression and the production of myocardial injury markers, ameliorated ventricular wall infarction, and reduced the levels of IL-1β, IL-6 and TNF-α via regulation of the PPAR γ/NF-κB pathway in the isoproterenol-evoked MI mice. Furthermore, it attenuated cell apoptosis and lightened the production of inflammation cytokines in lipopolysaccharide-stimulated human coronary artery endothelial cells (Li et al., 2018).

## Conclusion and Future Perspective

TCM has been used in China for thousands of years to treat various diseases. In recent decades, more and more studies have been carried out on TCM, and numerous impressive achievements have been achieved, such as artemisinin, a gift from TCM to the world (Tu, 2016). With the progress of biotechnology in component separation, more pharmacological active components have been isolated from TCM, and some of them have become commonly used drugs in clinic, such as berberine and curcumin. As an active ingredient of traditional herbal medicine Gegen, puerarin has been recognized to possess a wide range of pharmacological activities. Basic and clinical studies have indicated that puerarin is an effective therapeutic candidate against diabetes and its complications, osteonecrosis, cancer, Alzheimer’s disease, Parkinson’s disease and endometriosis. Furthermore, emerging studies over the past few decades have shown that puerarin plays a critical role in protecting humans and animals from CVDs, including atherosclerosis, cardiac hypertrophy, heart failure, diabetic cardiovascular complications, MI, stroke and hypertension. The efficacy of puerarin for treating multiple CVDs may be mainly mediated by modulation of Na^+^, K^+^ and Ca^2+^ channels, NF-κB, PI3K/Akt, BCL-2, and BAX. Furthermore, it may be related to the regulation of other targets such as PPAR, AMPK, AT1, ACE2, Nrf2, TNF-ɑ, IL-8, COX-2, and P2X_3_. Due to these cardiovascular protective effects, puerarin has been developed for clinical use. For example, Puerarin Injection, Puerarin and Glucose Injection, as well as Puerarin and Sodium Chloride Injection have been approved as clinical drugs for the treatment of certain CVDs in China. In other countries, a few clinical trials have been conducted to investigate the effect of puerarin on CVDs.

However, the solubility and permeability of puerarin are low, resulting in poor oral absorption and low bioavailability, which eventually limits its application in clinic. A simple increase in drug dose will not effectively improve bioavailability, and may cause side effects and toxicity. To overcome this obstacle, by modifying the structure of puerarin, a number of derivatives with better bioavailability have been developed, such as P1-EA, P2-EA, puerarin-7-*O*-glucuronide and 3′-methoxy puerarin. In addition, a variety of effective drug delivery systems of puerarin have been developed using nanotechnologies and multiple preparation technologies. Nevertheless, the toxicological data of puerarin derivatives and drug delivery systems are insufficient and need further research. Besides, other in-depth studies are required for puerarin development. The pathways underlying the cardiovascular properties of puerarin are complicated. Its direct molecular mechanism and key targets need to be further clarified. What’s more, further clinical trials with a larger sample size and rigorous design are needed to validate the current findings in the prevention and treatment of CVDs. With more intensive experimental and clinical studies on puerarin, its pharmacological mechanism will be revealed more definite, the forms of medication will be more diversified, and the clinical indications will be more extensive in the future. In this case, puerarin will also have the potential to become a safe and effective innovative Chinese medicine into the world.
